# A Large PROP1 Gene Deletion in a Turkish Pedigree

**DOI:** 10.1155/2018/2403430

**Published:** 2018-03-14

**Authors:** Suheyla Gorar, Doga Turkkahraman, Kanay Yararbas

**Affiliations:** ^1^Department of Endocrinology and Metabolism, Antalya Education and Research Hospital, 07100 Antalya, Turkey; ^2^Department of Pediatric Endocrinology, Antalya Education and Research Hospital, 07100 Antalya, Turkey; ^3^Department of Medical Genetics, Acibadem Mehmet Ali Aydinlar University, 34752 Istanbul, Turkey

## Abstract

Pituitary-specific paired-like homeodomain transcription factor, PROP1, is associated with multiple pituitary hormone deficiency. Alteration of the gene encoding the PROP1 may affect somatotropes, thyrotropes, and lactotropes, as well as gonadotropes and corticotropes. We performed genetic analysis of PROP1 gene in a Turkish pedigree with three siblings who presented with short stature. Parents were first degree cousins. Index case, a boy, had somatotrope, gonadotrope, thyrotrope, and corticotrope deficiency. However, two elder sisters had somatotroph, gonadotroph, and thyrotroph deficiency and no corticotroph deficiency. On pituitary magnetic resonance, partial empty sella was detected with normal bright spot in all siblings. In genetic analysis, we found a gross deletion involving PROP1 coding region. In conclusion, we report three Turkish siblings with a gross deletion in PROP1 gene. Interestingly, although little boy with combined pituitary hormone deficiency has adrenocorticotropic hormone (ACTH) deficiency, his elder sisters with the same gross PROP1 deletion have no ACTH deficiency. This finding is in line with the fact that patients with PROP1 mutations may have different phenotype/genotype correlation.

## 1. Introduction

The anatomical development of hypothalamic-pituitary-thyroid axis is completed during the first gestational trimester. Transcription factor genes playing a role in the development of hypothalamus and pituitary are pituitary transcription factor 1 (PIT1), Prophet of Pit-1 (PROP1), LIM Homeobox 3 (LHX3), LIM Homeobox 4 (LHX4), and HESX Homeobox 1 (HESX1) which all are known to be important for organ commitment and embryonic pituitary cell differentiation. In case of incomplete differentiation of anterior pituitary gland, one or more of these hormones may be affected, causing combined pituitary hormone deficiency (CPHD) [1]. This condition is mainly sporadic in occurrence, but familial forms have also been described with autosomal recessive, autosomal dominant, and X-linked recessive modes of inheritance. In familial or sporadic CPHD cases, the most common causes are PIT1 and PROP1 gene defects [2].

PROP1 gene is located on chromosome 5q35. Inactivating mutations in PROP1 perturb ontogenesis of pituitary gonadotrophs, somatotrophs, lactotrophs, and thyrotrophs. Somatotropic, thyrotropic, and gonadotropic function impairments manifests clinically as short stature, neonatal hypoglycemia, sequential loss of anterior pituitary tropic hormones [3].

Index case admitted to pediatric endocrinology clinic with complaint of short stature. His family history revealed presence of similar symptoms in his two siblings. Following hormonal examination of the cases, we conducted genetic analyses. PROP1 mutation screening detected a homozygous deletion of the entire PROP1 in three patients.

## 2. Case Presentation

### 2.1. Case 1: Index Case

A 12.3-year-old male patient was referred to our pediatric endocrinology clinic for evaluation of short stature. He has been using levothyroxine (LT4) for hypothyroidism for more than 2 years. In medical history, he was born at term weighing 3500 g with uneventful gestation and delivery. His parents were first degree cousins. The height of the mother and the father was 165.5 and 172 cm, respectively. He had three sisters and one brother. His brother and one of the elder sisters were healthy and 175 cm and 165 cm tall, respectively. On physical examination, height was 129 cm (SDS: −3.2) and weight was 28 kg (body mass index, BMI: 16.8, −1.0 SDS). Target height was 175.2 cm (SDS: −0.2). Testicular volume was 2 ml bilaterally with a 3 cm penile length. Bone age was 9 years. Laboratory findings revealed that free thyroxine (FT4) is 1.2 ng/dl (*N*: 0.61–1.57), thyroid stimulating hormone (TSH) is 0.01 *μ*IU/ml (*N*: 0.37–5), thyroid autoantibodies were negative, prolactin (PRL) is 4.5 ng/ml (*N*: 2.6–13.1), adrenocorticotropic hormone (ACTH) is 21.3 pg/ml (*N*: 4.7–48.8), cortisol is 6.8 *μ*g/dl (*N*: 6.7–22.6), and insulin-like growth factor 1 (IGF-1) is 12.8 ng/ml (*N*: 85.2–248.8). Thyroid ultrasonography revealed a hypoplasic thyroid gland (1.7 ml) with normal parenchyma. On pituitary magnetic resonance (MRI), partial empty sella was detected with normal bright spot (pituitary height was 2.8 mm). Clonidine and L.DOPA stimulated peak serum growth hormone (GH) levels were 2.1 ng/ml and 1.9 ng/ml, respectively. With these results diagnosis of GH deficiency was confirmed, and recombinant growth hormone (rGH) was initiated. On follow-up, low dose (1 *μ*g) ACTH stimulation test was performed, and adrenal deficiency was confirmed (peak cortisol: 12.1 *μ*g/dl). Then, oral hydrocortisone replacement therapy was initiated (10 mg/m^2^/day).

At 14.3 years, he was still prepubertal with testicular volume of 3 ml bilaterally. Basal level of testosterone was <0.01 ng/ml. Then, LHRH stimulation test was performed, and central hypogonadism was confirmed (peak luteinizing hormone, LH; 0.62 mIU/ml, and peak follicle stimulating hormone, FSH; 0.85 mIU/ml). Intramuscular depot form of testosterone was initiated, 50 mg/monthly.

### 2.2. Case 2: 3-Sister Siblings of Index Case

The 22- and 24-year-old females who are sisters of index case were referred to our endocrinology outpatient clinic. In their delivery history, they were delivered via spontaneous vaginal birth with uneventful gestation and delivery. They did not present hypoglycemia or respiratory distress during the neonatal period.

At the age of 13, junior sister was diagnosed with GH deficiency and received rGH and LT4 replacement therapy for 8 years but the hormonal examination resulting from that period could not be retrieved. Her height and weight measurements were 145 cm (−2.83 SDS) and 37 kg (BMI: 17.6, −1.7 SDS), respectively. She was 12 years old at the onset of menarche and was having irregular menstrual cycles with long periods of amenorrhea/oligomenorrhea. There was no axillary/pubic hair, and breast development was Tanner stage II. Her mental function was normal. Bone age was adult, and epiphyseal lines were closed. The ovaries were atrophic on pelvic USG. On pituitary MRI, partial empty sella was detected with normal bright spot (pituitary height was 1.5 mm). Her basal hormone levels are shown in Table 1. The patient did not respond to LHRH test (basal FSH was 0.01 mIU/ml and LH was 0.11 mIU/ml; peak FSH was 0.01 mIU/ml and peak LH was 0.12 mIU/ml). There was no response to thyrotropin releasing hormone (TRH) stimulation test (basal TSH 0.04 mU/L; peak TSH 0.03 mU/L). Although sufficient cortisol response was obtained in insulin-induced hypoglycemia test, no sufficient GH response was obtained. During hypoglycemia, peak cortisol level was 26 *μ*g/dL, while peak GH level was 0.01 ng/mL. The patient was put on LT4, conjugated estrogen, and adult-dose of rGH replacement therapy.

At the age of 15, elder sister was diagnosed with GH deficiency and central hypothyroidism and has received rGH and LT4 replacement therapy for 9 years. The hormonal evaluation results from that period could not be retrieved. Height was 154 cm (−1.43 SDS) and weight was 49 kg (BMI: 20.6, −0.3 SDS). Her age at onset of menarche was 12 years and her menstruation history was similar to that of her sister. Upon psychiatric evaluation, she had hard time in social interaction and self-expression, and she was found to have borderline intelligence with an Intelligence Quotient Test (IQT) score of 80. Her bone age was adult, and epiphyseal lines were closed. Ovaries were atrophic on pelvic USG. On pituitary MRI, partial empty sella was detected with normal bright spot (pituitary height was 2.5 mm). Basal hormone levels are given Table 1. The patient did not respond to the LHRH test (basal FSH 0.08 mIU/ml and LH 0.09 mIU/ml; peak FSH 0.09 mIU/ml and LH 0.11 mIU/ml). Additionally, there was no response to TRH stimulation test (basal TSH 0.01 mU/L; peak TSH 0.01 mU/L). During insulin-induced hypoglycemia test, maximum cortisol level was measured as 19.5 mg/dL, and growth hormone level was 0.01 ng/mL. With these findings, the case was put on LT4, conjugated estrogen, and adult doses of rGH replacement therapy.

### 2.3. Genetic Analysis

Genomic DNA of the family members was extracted according to the manufacturer's standard procedure using the QIAamp DNA Blood Midi Kit (Qiagen, Hilden, Germany). The DNA samples were quantified with a nanophotometer (Implen, Germany) and used at a concentration of 50 ng/*μ*L. PROP1 gene was amplified using PCR primers: Forward (5′ ACCTACACACACATTCAGAGAC 3′), Reverse (5′ TGGAGCCTATGCTTTCAGC 3′), Forward (5′ AAAGACTGGAGCAGCACAGG3′), Reverse (5′ GGTGGTGAGATGAGGCCTGT 3′), and Forward (5′ GCCTTGTGGAAGAGCTTTACTCC 3′), Reverse (5′ CACCATGCATCTGCTTCACCC 3′). PCRs were validated by using 2% agarose gel electrophoresis (Fermentas, Lituenia). PCRs for each individual were mixed to obtain PCR pools, purified and quantified.

**Figure 1:**
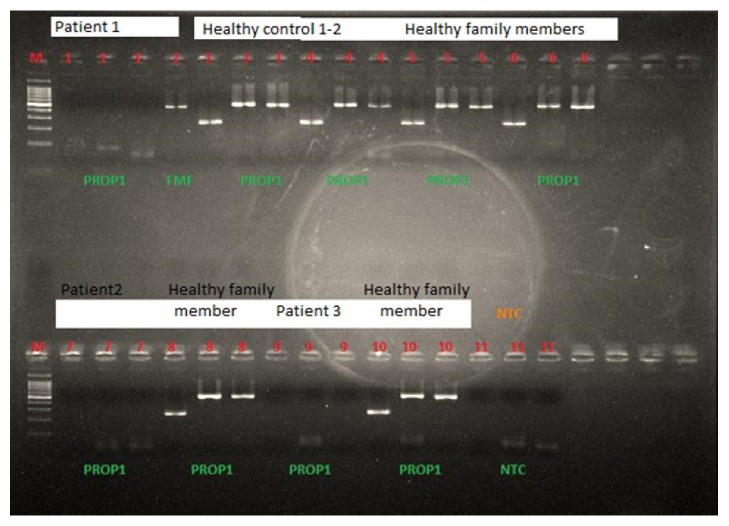
Three patients showed no amplification in PROP1 locus, whereas reference gene (Mediterranean fever-MEFV) was amplified. Healthy controls and healthy family members showed successful amplifications either.

Purifications were done by using exosap purification program (ExoSAP-IT, Affymetrix Inc., USA). Second Sephadex column (Sigma, Germany) was used for the PCR purification. Gel electrophoresis revealed no amplification of the gene, so in order confirm a possible gross deletion, healthy controls were tested for the same gene, as well as healthy family members. In addition Mediterranean fever (MEFV) gene amplification was performed simultaneously as reference amplification. MEFV amplification was successful in all patients. On behalf of these results, gross deletion involving PROP1 coding region was concluded as disease causing mutation in these patients (Figure 1).

## 3. Discussion

Genetic aetiologies of isolated pituitary hormone deficiency or CPHD have been researched for many years. CPHD occurs due to recognized mutations of transcription factors such as HESX1, PROP1, POU1F1, LHX3, and LHX4. PROP1 mutations represent the most common known genetic defect of both familial and sporadic CPHD. Phenotypic characteristics may be variable in CPHD result from these transcription factors' mutations, including PROP1. Published case reports, population studies, and reviews on genetic analysis of CPHD have shown new genetic variations, differences in the severity of the hormonal deficits, and the time of onset. As a result from clinical and hormonal phenotype highly variable [4–6], general properties of PROP1 mutation are a clinical disorder where GH deficiency is observed together with one or more anterior pituitary hormone deficiencies. The main clinical symptom is growth retardation with an onset during infancy or early childhood. Hypothyroidism is often mild and develops during late infancy or childhood. The affected individuals are not expected to be infertile, but their secondary sexual development can be delayed and incomplete. Untreated men generally have smaller testes and penis. Some of the affected women have menstrual bleeding, but may often require hormone replacement therapy afterwards. ACTH deficiency is less common but may also be seen, which usually develops during adolescence or adulthood [6]. PROP1 deficiency was first discovered among Ames mice which clinically results in dwarf mice with CPHD. The most common pituitary hormone deficiencies are of GH, TSH, FSH, LH, and PRL, while ACTH deficiency is also observed, though rarely [3].

It was shown that deletion of PROP1 in mice causes severe pituitary hypoplasia with failure of the entire PIT1 lineage and delayed gonadotrope development. Pituitary hormone deficiencies caused secondary endocrine problems and a high rate of perinatal mortality due to respiratory distress. Lung atelectasis in mutants correlated with reduced levels of NKX2.1 (TITF1; 600635) and surfactant (SFTPA1; 178630). Lethality of mice homozygous for either the null allele or a spontaneous hypomorphic allele was strongly influenced by genetic background [7]. As further human studies included familial total deletions of the gene all causing CPHD phenotype, gross deletions became well characterized in Human Gene Mutation Database (HGMD) as disease causing mutations [2, 8–11]. PROP1 spans less than 4 kb of genomic DNA; no benign copy number or deletion variations are defined in Decipher or Genomic Variant Database (DGV). The extent of the reported deletions was variable but all totally covered the PROP1 gene. This is the sixth report of gross PROP1 deletion worldwide. No further information about the deletion is available in this family since high resolution array was not performed. Deletions were detected by PCR gel electrophoresis. Amplification was obtained in obligate heterozygote carriers and since the reactions were performed with reference genes and healthy controls, no further confirmatory test was performed.

In this study, we presented the hormonal and genetic evaluations of three siblings having PROP1 deletion. Interestingly, although little boy with CPHD has ACTH deficiency, her elder sisters with the same gross PROP1 deletion have no ACTH deficiency. Peak cortisol levels of elder sisters were considered adequate in insulin-induced hypoglycemia test [12, 13]. Also, they were stable in terms of clinical and biochemical status. But, clinical and hormonal evaluations of cases were continued follow-up because of possible adrenal insufficiency in adulthood period. This finding is in line with the fact that patients with PROP1 mutations definitely may have different phenotype/genotype correlation. Deladoëy et al. [14] studied 36 families with a total of 73 affected patients with CPHD. They demonstrated, based on a great variability in phenotype, the secretion of pituitary-derived hormones (GH, TSH, LH, and FSH) decline gradually with age, following a different pattern and time scale in each individual. On the other hand, seven patients presented low basal levels of cortisol and ACTH, but stimulated levels after insulin-induced hypoglycemia revealed no abnormality. None of the patients were on cortisol replacement therapy. Similarly, et al. [15] showed that two siblings with PROP1 mutation who presented with short stature have not corticotropin deficiency. In a multicentric study conducted by Vallette-Kasic et al. [16] in France, 27 unrelated families originated from five different countries screened for PROP1 gene anomalies. Patients were included on the basis of GH deficiency associated with at least one other pituitary hormone deficiency. Cortisol was initially found normal in all patients, but late onset ACTH deficiency was observed in four patients. In insulin-induced hypoglycemia test, two patients were blunted cortisol response and given cortisol replacement therapy. As a result, they emphasized that corticotroph deficiency was frequently observed in association with GH, TSH, and gonadotropin deficiency in PROP1 gene alteration and should be carefully sought during follow-up. In another study, discussed by Reynaud et al. [17], genetic screening was performed in 195 patients with combined pituitary hormone deficiency. In 109 patients without extrapituitary abnormalities, 20 had PROP1 mutations, including eight patients with a family history of CPHD. Eighteen of 20 patients carried PROP1 mutations had gonadotroph and somatotroph deficiency at postpubertal age, while two patients had corticotroph, thyrotroph, and somatotroph deficiency at pubertal age.

In conclusion, PROP1 gene has maintenance role in five types of principal anterior pituitary hormone-secreting cells, which are somatotroph, lactotroph, thyrotroph, gonadotroph, and corticotroph, basis, and their differentiation progress. But, in human trials different mutations that cause CPHD in the PROP1 gene have been shown [2, 5]. Variability of mutations and deletions in PROP1 gene can cause different types of hormone deficiency. This state is very important for therapy and follow-up of the patients.

## Figures and Tables

**Table 1:** Baseline hormone levels of sister siblings.

Hormone (normal range)	Sibling 1	Sibling 2
ACTH (4,5–48 pg/ml)	12,5	7,5
Kortizol (6,7–22,6 ug/dl)	9,82	2,34
FSH (1,2–19,1 mlU/ml)	0,01	0,08
LH (1,24–8,6 mlU/ml)	0,01	0,04
TSH (0,34–5,86 ulU/ml)	0,01	0,03
FT4 (0,61–1,12 ng/dl)	1,10	0,99
Prolactin (2–15 ng/ml)	9,12	4,43
GH (0,003–0,971 ng/ml)	0,02	0,01
IGF-1 (135–449 ng/ml)	4,11	10,62

ACTH: adrenocorticotropic hormone; FSH: follicle stimulating hormone; LH: luteinizing hormone; TSH: thyroid stimulating hormone; FT4: free thyroxine; GH: growth hormone; IGF-1: insulin-like growth factor 1.
